# Simultaneous Two-Photon Voltage or Calcium Imaging and Multi-Channel Local Field Potential Recordings in Barrel Cortex of Awake and Anesthetized Mice

**DOI:** 10.3389/fnins.2021.741279

**Published:** 2021-11-11

**Authors:** Claudia Cecchetto, Stefano Vassanelli, Bernd Kuhn

**Affiliations:** ^1^Department of Biomedical Sciences, Section of Physiology, University of Padua, Padua, Italy; ^2^Optical Neuroimaging Unit, Okinawa Institute of Science and Technology Graduate University, Okinawa, Japan; ^3^Padua Neuroscience Center, University of Padua, Padua, Italy

**Keywords:** voltage imaging, local field potentials, ANNINE-6, two-photon microscopy, neuroimaging, combined approach, whisker stimulation, cortex

## Abstract

Neuronal population activity, both spontaneous and sensory-evoked, generates propagating waves in cortex. However, high spatiotemporal-resolution mapping of these waves is difficult as calcium imaging, the work horse of current imaging, does not reveal subthreshold activity. Here, we present a platform combining voltage or calcium two-photon imaging with multi-channel local field potential (LFP) recordings in different layers of the barrel cortex from anesthetized and awake head-restrained mice. A chronic cranial window with access port allows injecting a viral vector expressing GCaMP6f or the voltage-sensitive dye (VSD) ANNINE-6plus, as well as entering the brain with a multi-channel neural probe. We present both average spontaneous activity and average evoked signals in response to multi-whisker air-puff stimulations. Time domain analysis shows the dependence of the evoked responses on the cortical layer and on the state of the animal, here separated into anesthetized, awake but resting, and running. The simultaneous data acquisition allows to compare the average membrane depolarization measured with ANNINE-6plus with the amplitude and shape of the LFP recordings. The calcium imaging data connects these data sets to the large existing database of this important second messenger. Interestingly, in the calcium imaging data, we found a few cells which showed a decrease in calcium concentration in response to vibrissa stimulation in awake mice. This system offers a multimodal technique to study the spatiotemporal dynamics of neuronal signals through a 3D architecture *in vivo*. It will provide novel insights on sensory coding, closing the gap between electrical and optical recordings.

## Introduction

Two-photon microscopy and electrophysiology are complementary techniques to detect neuronal activity *in vivo* with high spatial and high temporal resolution, respectively.

Two-photon microscopy (Denk et al., 1990; Theer et al., 2005) allows optical sectioning and overcomes, to some extent, the problem of light scattering inherent to imaging of biological tissue. In combination with functional dyes, it allows *in vivo* imaging of neuronal activity from subcellular compartments to whole brain regions. A wide spectrum of dyes for converting biological processes into fluorescent signals are available. Most popular ones are calcium indicators, synthetic or genetically encoded, which allow to image suprathreshold neuronal activity like trains or bursts of action potentials (APs) (Russell, 2011; Lin and Schnitzer, 2016). Typically, subthreshold events are not detected, and the responses are relatively slow (up to seconds) compared to the electrical neuronal activity and the high temporal resolution of electrophysiology. However, also a number of voltage-sensitive dyes (VSDs) were shown to be compatible with two-photon microscopy, providing a highly resolved and direct measure of the membrane potential. Here, we focus on the pure electrochromic VSD family of ANNINE-6 (Hübener et al., 2003; Kuhn and Fromherz, 2003; Kuhn and Roome, 2019), and specifically ANNINE-6plus (Fromherz et al., 2008), which shows high fractional signal and negligible bleaching and phototoxicity when excited at the red spectral edge of absorption (Kuhn et al., 2004; Kuhn and Roome, 2019; Roome and Kuhn, 2019). If single neurons are labeled with ANNINE-6plus and imaged with two-photon microscopy, spatio-temporal resolutions of 5 μm per 5 ms or 25 μm per 1 ms for a 10 mV voltage changes in Purkinje neuron dendrites can be achieved (Roome and Kuhn, 2018). When ANNINE-6 dyes are bulk loaded into neuronal tissue, the outer leaflet of all cell membranes will be labeled and, therefore, the average membrane voltage of the excitation volume is measured (Kuhn et al., 2008; Dalphin et al., 2020). Due to the intermingled structure of brain tissue, processes from many different cells and cell types (neurons and glia) contribute to the recorded signal. This signal mixing reduces the spatio-temporal resolution dramatically, but still allows to image voltage fluctuations averaged in space and/or time.

Electrical recordings with implanted neural probes, on the other hand, can achieve excellent temporal resolution, i.e., up to several kHz, covering the range of extracellular APs (Fiáth et al., 2019; Mahmud et al., 2020). Each electrode, typically only a few micrometers in width, samples the extracellular voltage activity coming from a population of neurons in the proximity of its surface (Buzsáki, 2004; Obien and Frey, 2019). Up to hundreds or even thousands recording sites can be arranged into a multielectrode array, e.g., on the tip of a probe, having one or more shanks (Schroder et al., 2015; Scholvin et al., 2016; Jun et al., 2017). CMOS-based neural probes with 256 multiplexed recording channels are able to establish a strong capacitive coupling with the brain tissue, forming an excellent capacitive interface for recording cortical local field potentials (LFPs) with 10–15 μm spatial resolution in the barrel cortex (Cecchetto et al., 2015; Schroder et al., 2015). Moreover, intracortical microelectrode arrays with metal electrodes, such as the commercially available Atlas Neuroengineering probes^1^, enable the possibility to read small amplitude signals in the tens-microvolts to millivolts range, thus detecting fine electrophysiological traits such as single APs and multi-unit activity (MUA). For example, APs and MUA with amplitudes that can reach up to 150 μV could be recorded with a noise of about 10 μV RMS from the rat barrel cortex (Tambaro et al., 2021). Similarly, in the lower frequency range, LFPs and also low-frequency oscillations were reliably recorded with the same probe in the hippocampus of transgenic AD mice (Leparulo et al., 2020).

Combining these two methods is complicated by the steric hindrances as the space under the objective is very limited, especially when using objectives with high numerical aperture which are critically important for two-photon microscopy. Additionally, probes are typically made of non-transparent materials such as silicon, gold, and iridium, and therefore will partially or completely block the excitation light if placed inside or above the imaging field. Moreover, light-induced artifacts generated by photovoltaic, photoelectric, and photothermal effects in the probes contaminate electrical and optical recordings (Seemann and Kuhn, 2014; Thunemann et al., 2018).

Transparent graphene or bilayer nanomesh microelectrode arrays were previously used with simultaneous deep two-photon imaging and optogenetic manipulations in primary somatosensory cortex and visual cortex (Qiang et al., 2018; Thunemann et al., 2018). Nevertheless, these microarrays are laid directly on the brain surface and cannot sample electrical signals from regions deeper than about 90 micrometers [estimated distance using CMOS-based microelectrodes array, (Frey et al., 2009)], thus preventing depth-resolved electrical recordings. Moreover, planar array implants often require dura mater removal, which can cause inflammation and partial rearrangement of the neural connections on the very superficial portion of layer 1. Finally, multi-electrode array recordings were previously combined with wide field voltage imaging (Tominaga et al., 2001), but unfortunately wide field imaging lacks the spatial resolution of two-photon microscopy.

The rodent barrel cortex represents an ideal brain region to test simultaneous two-photon voltage or calcium imaging and electrical recordings, given the characteristic and distinctive evoked responses that can be elicited by vibrissa stimulation in this area (Petersen et al., 2003; Fox, 2008; Kuhn et al., 2008). The mouse somatosensory cortex is a well-known example of topographic mapping where each of the whisker on the snout of the animal is specifically mapped onto a cortical area called barrel. Barrels can be found in layer IV (the main input layer of the somatosensory pathway) and are separated from each other by narrower zones called septa (Woolsey and Van der Loos, 1970; Land and Simons, 1985). The barrel cortex forms an early stage of cortical processing for tactile information, along with the trigeminal ganglion and the thalamus.

Here, we used two-photon voltage or calcium imaging combined with simultaneous multi-channel electrical recordings to record spontaneous and stimulus-evoked activity from the primary somatosensory cortex of anesthetized and awake mice. A 32-channels implantable neural probe was inserted into the cerebral cortex of mice through an access port of the chronic cranial window (Roome and Kuhn, 2014, 2019). This allowed us to record electrophysiological activity from multiple cortical layers while performing depth resolved voltage or calcium imaging.

## Materials and Methods

All animal experiments were approved by the OIST Institutional Animal Care and Use Committee (IACUC) in an Association for Assessment and Accreditation of Laboratory Animal Care (AAALAC International) accredited facility.

### Surgery

Chronic cranial window surgeries [for detailed protocol see Augustinaite and Kuhn (2020a)] were performed on 67- to 129-days old male C57/BL6 mice using a 5 mm glass cover slip with off-center silicone access ports as described previously (Roome and Kuhn, 2014, 2019). Mice were deeply anesthetized during the surgery by a 2% isoflurane induction followed by a mixture of medetomidine (0.3 mg/kg), midazolam (4 mg/kg), and butorphanol (5 mg/kg) injected intraperitoneally. Eye gel was applied to the eyes for their protection and to prevent dehydration. Carprofen (5 mg/kg, intraperitoneal) and Buprenorphine (0.1 mg/kg, subcutaneous) were administered to reduce post-operative inflammation and pain. Dexamethasone (2 mg/kg, intramuscular) was also administered to reduce inflammation. Hair was removed with a trimmer and hair removal cream. The skin was then opened with a scalpel and the connective tissue was removed with a cotton tip and forceps. The skull was cleaned with a 2% lidocaine solution and dried with compressed air. Using a dental drill, the area around the intended craniotomy (center at 1.5 mm posterior of bregma, 2.5 mm lateral, diameter 5 mm) was thinned. A wooden toothpick was attached vertically to the center of the intended craniotomy region using super glue. After a few minutes, once the glue was set, the toothpick was carefully lifted to separate the skull from the dura and then to remove the bone. The glass cover slip was mounted onto the dura with the 1-mm diameter silicone access port toward anterior direction and sealed with super glue to the skull. For ECoG recordings, one small hole was drilled into the skull on both hemispheres and in each a silver wire (0.404 mm thick, 3 cm long, with one end flattened by hammering) was inserted between the skull and the dura and then glued with superglue. A rectangular aluminum headplate with 6-mm opening was positioned over the window and attached with dental acrylic, covering all areas of exposed skull. At the end of the surgery, saline was injected (500 μl, subcutaneous) to avoid dehydration of the animal. Atipamezole hydrochloride (0.3 mg/kg, intraperitoneal) was administered to allow the mouse to wake up from anesthesia.

### Injection Through the Access Port

For voltage imaging experiments an ANNINE-6plus labeling solution was prepared [for detail protocol see Roome and Kuhn (2019)]: a stock solution of ANNINE-6plus (Dr. Hinner and Dr. Hübener Sensitive Farbstoffe GBR, Munich, Germany^2^; info@sensitivefarbstoffe.de) was prepared in DMSO (Nacalai Tesque) with 20% Pluronic F-127 (AAT Bioquest) at a concentration of 2.0 mM (200 μg of ANNINE-6plus in 140 μl DMSO with 20% Pluronic F-127). This stock solution was kept up to a year at room temperature protected from light. Before use, the stock solution was heated to 70°C in a heating block for at least 30 min. The heated ANNINE-6plus stock solution was then diluted to 5% in saline (0.9% NaCl in H_2_O) and immediately filled into the injection pipette.

After 11–16 days of recovery, for calcium imaging experiments, an Adeno-Associated Viral vector (AAV) for GCaMP6f expression (AAV1.Syn.GCaMP6f.WPRE.SV40, addgene) or, for voltage imaging experiments, ANNINE-6plus were injected through the silicone access port at the two-photon microscope setup, using a 20 to 30-degree beveled glass pipette with an opening between 5 and 10 μm. The pipette entered the port at an angle of 27°. AAV was injected 300 and 600 μm below the dura, and ANNINE-6plus was injected 400 μm below the dura. AAV was injected at a rate of 10 nl/min, with a total amount of 140 nl for each injection depth. ANNINE-6plus delivery was performed slowly and carefully, aiming for about 700 nl of dye solution being delivered over 1 h.

A total of *n* = 10 mice were injected, *n* = 6 with ANNINE-6plus and *n* = 4 with the AAV for GCaMP6f expression.

### Imaging Setup

We used a custom-built combined wide-field and two photon microscope (MOM, Sutter) with a 5×/N.A. 0.13 air objective (Zeiss) or a 25×/N.A. 1.05 water immersion objective with 2 mm working distance (Olympus). A femtosecond-pulsed Ti:sapphire laser was used to excite fluorescence which was detected by two GaAsP photomultiplier tubes (Hamamatsu) in the spectral range of 490–550 nm (green) and 550–750 nm (red), separated by a dichroic mirror at 552 nm (all Semrock). The microscope was controlled by commercial software (MScan, Sutter Instruments).

### Imaging

Calcium imaging was recorded with 512 × 512 pixels full-frame scans. The field of view was 375 × 375 μm^2^ and the sampling frequency 30.9 Hz. For voltage imaging, box-scans with 512 × 32 pixels (375 × 24 μm^2^, 500 Hz) and 512 × 16 pixels (375 × 12 μm^2^, 1000 Hz) were acquired. By underfilling the back aperture of the objective and turning the collar of the 25× objective to highest imaging depth, the point spread function of excitation was extended to 5 μm [see Supplementary Figure 10 in Roome and Kuhn (2018)]. For every imaging depth and box-scan type, 10 min long scans were acquired. One epoch was taken with no stimulations (i.e., spontaneous activity), while the following was taken while multi-whisker air-puff stimuli (100 ms, 20 psi) were delivered to the contralateral whisker pad with random timing and average interstimulus interval of 10 s. Images were acquired both during light anesthesia (1% isoflurane) and wakefulness (resting or running). At the beginning of the experiment and right after probe insertion, z-stacks (total travel of 540 μm with 2 μm z-steps, 50 averages per plane) were acquired. During anesthesia, the mouse body temperature was monitored and kept at 37°C by means of a heating pad equipped with a rectal probe. Sleep spindles were visualized and checked in the ECoG traces during the acquisitions, and the absence of reflexes was checked. Breath rate was regular, with no episodes of difficult breathing (which instead can happen for high doses of isoflurane or in very deep anesthesia regimes). Awake recordings followed, after recovery from anesthesia for 30–40 min. To confirm that the mouse fully recovered from anesthesia, the ECoG trace was used to confirm de-correlated LFP activity. Further, the presence of active whisking and motor activity were also used to confirm full wakefulness of the mouse. During wakefulness, movements of the cylindrical treadmill were recorded through a rotary encoder (E6A2-CW3C, OMRON).

### Electrophysiology

Electrophysiological signals were acquired by a silicon-based probe with a linear matrix of 32 electrodes (ATLAS Neuroengineering Probe: E32+R-50-S1-L10 NT; pointy tip feature; IrOx electrodes; spacing between electrodes: 50 μm) and connected via an SPI cable to the acquisition system (Open Ephys, OEps Tech). The acquisition board was equipped with an I/O board for interfacing with auxiliary devices: the air-puff TTL and the frame sync signal from the two-photon microscope were acquired and used to synchronize the electrophysiological signal with the stimulation and the image acquisition. While the mouse was anesthetized, the Atlas probe was inserted into the tissue at a depth of 850 μm under an angle of 27° to the cranial window under visual control using an sCMOS camera (PCO.edge 4.2, PCO) under white light illumination. The probe was inserted using a micromanipulator (Sutter MP-285) at slow speed (30–50 μm/s). Particular attention was given to inserting the probe with the side containing the array facing downward, i.e., away from the excitation laser beam, to minimize the amount of light impinging on the electrodes. The LFP signal was visualized, recorded, and digitalized at 10 kHz through an open-source software interface supplied with the acquisition system.

The electrocorticogram (ECoG) was recorded at 1 kHz through the silver wires implanted during the surgery using an EEG pre-amplifier (100 × gain, sigmann elektronik GmbH) with additional band-pass filtering (0.5–200 Hz; Model 440, Brownlee precision).

### Data Analysis

Movies were analyzed with custom MATLAB code and ImageJ. ROI selection from calcium imaging data was done in ImageJ. Image registration and motion correction were done in MATLAB using a fast variational method (Flotho et al., 2021).

Electrophysiological signals were converted from .continuous filetype (Open Ephys output files) to .mat files and analyzed through custom MATLAB routines.

Signals acquired by the imaging and electrophysiology setups were synchronized based on the frame sync and TTL signals.

### Histology

Mice were transcardially perfused at the end of the experiments with 4% periodate-lysine-paraformaldehyde (PLP) (McLean and Nakane, 1974), brains were extracted and further fixed in PLP. Fixed brains were cut in 100-um thick sagittal slices. Images were taken with a widefield fluorescence microscope (Nikon, Eclipse Ni-E, 10×/0.45 Plan Apo objective) equipped with a DS-Qi1Mc camera.

## Results

### Two-Photon Voltage or Calcium Imaging Combined With Simultaneous Metal-Electrode Array Recording

**FIGURE 1:**
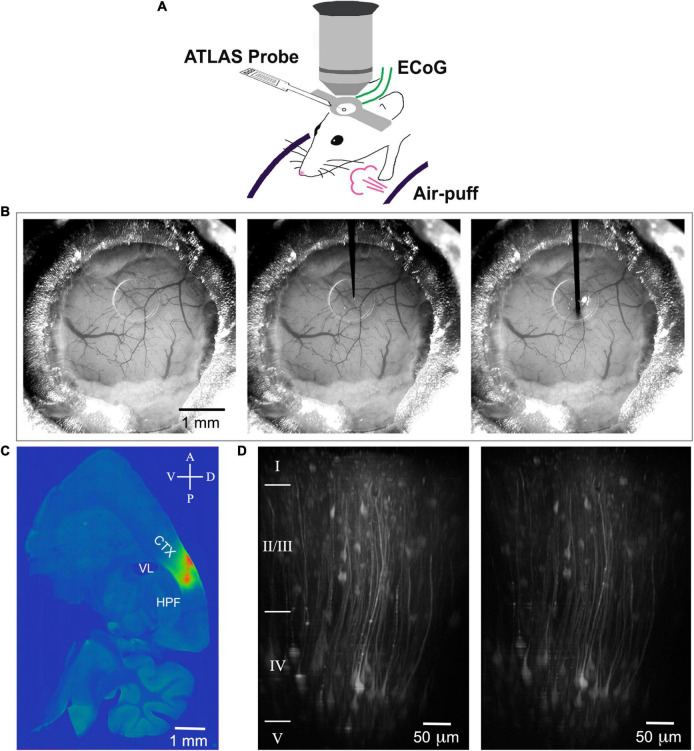
Scheme of the experimental setup, probe insertion and reconstruction of the insertion track. **(A)** A mouse is head-fixed on a cylindrical treadmill. An electrical probe (Atlas Neuroengineering) is inserted through the access port of the chronic cranial window into the brain and allows electrical recording. The window allows two-photon imaging of voltage or calcium depth resolved. For sensory stimulation, air puffs can be applied. **(B)** Mouse cranial window before probe insertion (left), with the tip of the probe positioned on the surface of the silicon plug (center), and with the probe inserted 850 μm deep into cortex (right). **(C)** ANNINE-6plus fluorescence in a 100-μm thick sagittal brain slice (LM ≈ 2.3 mm). The pipette for loading ANNINE-6plus and the electrical probe are entering through the same port under the same angle. Therefore, the track of the probe insertion is very well approximated by the track of ANNINE-6plus labeling. CTX: cortex, somatosensory areas; VL: lateral ventricle; HPF: hippocampal formation **(D)** Two-photon reconstruction of cortical neurons expressing GCaMP6f before (left) and after (right) probe insertion with tip below imaging site. Imaging was done at the center of the craniotomy, right below the access port (AP 1.5 mm; LM 2.5 mm). Cortical layers were identified based on (DeFelipe, 2002) and indicated on the left of the image.

After the mouse recovered from surgery for at least 10 days, ANNINE-6plus was injected with a beveled quartz pipette through the silicone access port of the chronic cranial window. This procedure was performed with a micromanipulator under the two-photon microscope, but with low magnification wide field observation. We waited for 12 h to make sure the DMSO is washed out. We used the same micromanipulator to mount the Atlas probe and entered under the same angle, and, if possible, through the same hole in the silicone access port (Figures 1A,B). It was important to choose a shallow angle to approach the chronic cranial window with access port as the working distance of the 25× objective used for two-photon imaging is only 2 mm, the front lens is 6.2 mm, and the tapering angle of the objective is 34°. Here, we chose an approach angle of 27°. The insertion speed was between 30 and 50 μm/s. Due to its sharp tip, the insertion of the probe did not cause bleeding or affect the tissue. Severing or dragging of the microvasculature was not observed on the cortical surface (Figure 1B) and tissue deformation was minimal along the insertion track (Figure 1C). Care was taken that the metal electrodes are facing downward so that they are not exposed to the incoming femtosecond pulses of the laser. The tip of the Atlas probe was lowered to a depth of about 850 μm corresponding to layer 5 or 6 in barrel cortex, as indicated by the previously injected ANNINE-6plus (Figure 1C). Finally, it was possible to simultaneously image voltage with two-photon microscopy and record electrophysiological signals with an electrode array.

**FIGURE 2:**
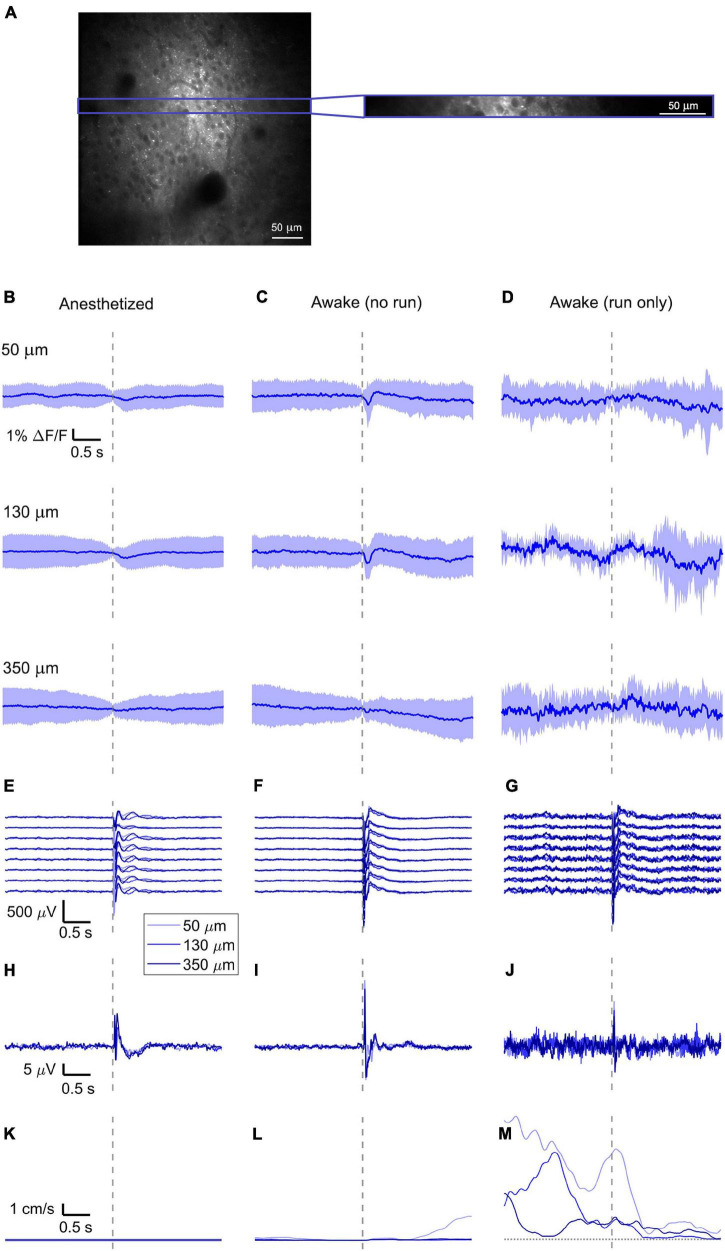
Simultaneously recorded average VSD signal and LFP, both in barrel cortex, ECoG, and running speed in response to a facial air puff (one mouse). **(A)** A representative imaged cortical region: 375 × 375 μm^2^ full-frame scan and correspondent 375 × 24 μm^2^ box-scan imaged at 130 μm deep in the cortex. **(B–D)** VSD responses evoked by an air puff to the vibrissae and cheek of the mouse recorded in 375 × 24 × 5 μm^3^ and 375 × 12 × 5 μm^3^ box-scans from a single mouse at three different depths **(B)** during anesthesia (50 μm: 139 trials, 130 μm: 139 trials, and 350 μm: 138 trials), **(C)** in awake mouse during resting (50 μm: 127 trials; 130 μm: 125 trials; and 350 μm: 121 trials), and **(D)** in awake mouse during running (50 μm: 7 trials; 130 μm: 8 trials; and 350 μm: 12 trials). **(E–G)** LFP recorded in a single mouse by eight electrodes (every 4th channel of the linear probe, corresponding to a distance of 200 μm along the probe, Δdepth = 68 μm between adjacent traces). The probe was inserted with the tip at 850 μm below the dura. Each channel shown is the average of the same number of trials as for the imaging (light blue at 50 μm, blue at 130 μm, and dark blue at 350 μm). The bottom LFP corresponds to the electrode located 800 μm below the dura, the top LFP to the electrode located 100 μm below the dura. Corresponding **(H–J)** average ECoG and **(K–M)** average running speed. Dashed gray lines indicate the stimulus onset. All traces are plotted as mean ± standard deviation.

The bulk loading of the brain tissue with ANNINE-6plus resulted in strong fluorescence in an area of about 300 μm. Somata were recognized as dark shadows (Figure 2A). For voltage imaging we recorded high speed box-scans (512 × 32 pixels at 500 Hz and 512 × 16 pixels at 1 kHz) from the left hemisphere of *n* = 6 mice for 600 s at every imaging depth, with one air puff delivered every 10 s (on average, random interstimulus intervals) as sensory stimulus to the contralateral, right vibrissae and cheek of the mouse. We chose an excitation wavelength of 1020 nm for voltage imaging to keep bleaching and phototoxicity to a minimum. The imaging power at the sample was comprised between 25 mW (for superficial layers) and 50 mW (for the deepest imaged layers). The laser power is higher than for typical calcium imaging because of the ANNINE-6plus excitation close to the red spectral edge of absorption (Kuhn et al., 2004; Kuhn and Roome, 2019). We were able to image voltage to a depth of 380 μm.

Alternatively, we injected in *n* = 4 mice an AAV delivering the gene of the genetically encoded calcium indicator GCaMP6f, as genetically encoded dyes result in high contrast labeling of genetically targeted cell types and, therefore, allow detailed reconstructions with two-photon microscopy and reliable recordings of calcium signals. The AAV was injected through the access port of the chronic cranial window and we allowed 15–18 days for the expression of GCaMP6f in neurons of layer 2/3 and 5 (Nathanson et al., 2009). Before and after insertion of the Atlas probe, neurons were reconstructed down to layer 5 (Figure 1D and Supplementary Figure 5). For calcium imaging we recorded 512 × 512 pixels images at 30.9 Hz for 600 s and the same stimulus as in ANNINE-6plus recordings. For excitation we used a wavelength of 950 nm. The laser power below the objective was between 10 mW (for superficial layers) and 50 mW (for the deepest imaged layers). Experiments were repeated in anesthetized and awake mice.

Simultaneously to the imaging, LFPs were acquired at 10 kHz with an Atlas probe having 32 electrodes. The tip of the probe was inserted 850 μm deep from the dura. Thus, the first electrode of the linear array was located below the imaging plane at a typical depth of 800 μm. To reduce noise, we averaged the signals of all trials. No photo- or thermal-induced effects were observed in the LFP traces, regardless of the laser wavelength or power used for imaging. Also, no artifacts in the imaging data were observed.

For simplicity and proof of principle, we recorded voltage and calcium separately using two distinct groups of mice. It was previously shown (Roome and Kuhn, 2018) that it is possible to measure both simultaneously.

### Average Optical and Electrical Voltage Responses

To reduce biological and optical noise, we averaged the full box-scan, resulting in a single trace representing the average membrane voltage changes at a specific depth in a 375 × 24 × 5 μm^3^ or 375 × 12 × 5 μm^3^ box volume and averaged all trials sorted into three behavioral states (anesthetized, awake but resting, and running) and three imaging depths (50, 130, and 350 μm) (example of one mouse, Figures 2B–D).

**FIGURE 3:**
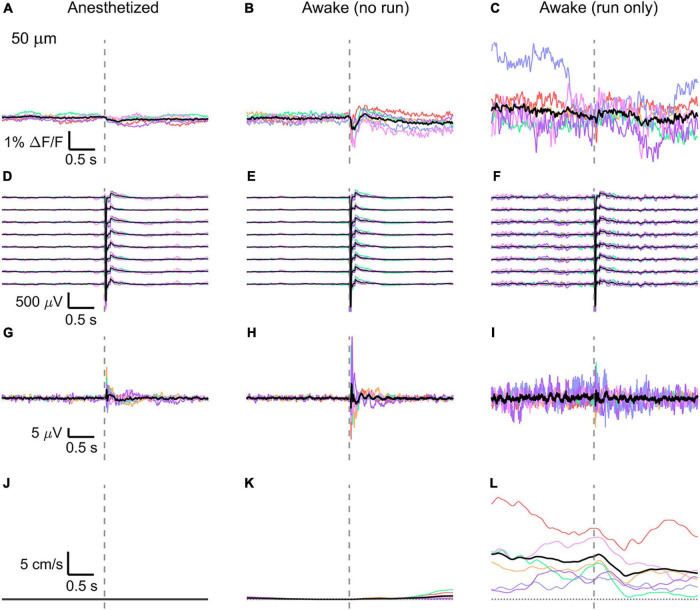
Average VSD signal 50 μm below dura and LFP, both in barrel cortex, ECoG, and running speed in response to a facial air puff (six mice). Average VSD responses evoked by an air puff to the vibrissae and cheek of the mouse in layer I (50 μm below dura) recorded in 375 × 24 × 5 μm^3^ and 375 × 12 × 5 μm^3^ box-scans in six mice **(A)** during anesthesia (133, 187, 139, 139, 126, 134 trials), **(B)** in awake mice during resting (111, 165, 118, 127, 104, 123 trials), and **(C)** in awake mice during running (16, 27, 14, 7, 16, 11 trials). **(D–F)** Corresponding average LFP recorded by eight electrodes (every 4th channel of the linear probe, tip at 850 μm below dura, bottom LFP trace: 800 μm, top LFP trace: 100 μm below dura, Δdepth = 68 μm between adjacent traces). Corresponding **(G–I)** average ECoG and **(J–L)** average speed. Each mouse is indicated by a different color; the overall average is shown in black. Gray dashed line marks the air puff onset.

**FIGURE 4:**
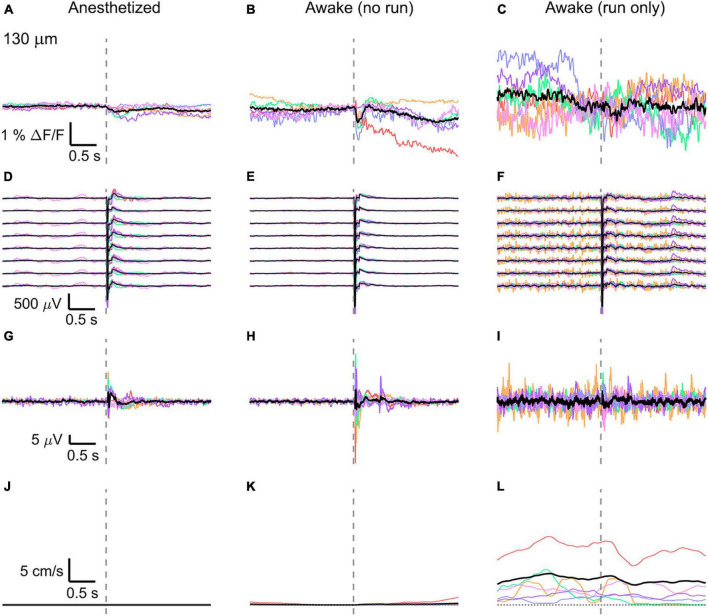
Average VSD signal 130 μm below dura and LFP, both in barrel cortex, ECoG, and running speed in response to a facial air puff (six mice). Average VSD responses evoked by an air puff to the vibrissae and cheek of the mouse in layer II (130 μm below dura) recorded in 375 × 24 × 5 μm^3^ and 375 × 12 × 5 μm^3^ box-scans in six mice **(A)** during anesthesia (135, 187, 139, 139, 130, 138 trials), **(B)** in awake mice during resting (109, 195, 125, 125, 96, 124 trials), and **(C)** in awake mice during running (19, 1, 7, 8, 13, 11 trials). **(D–F)** Corresponding average LFP recorded by 8 electrodes (every 4^th^ channel of the linear probe, tip at 850 μm below dura, bottom LFP trace: 800 μm, top LFP trace: 100 μm below dura, Δdepth = 68 μm between adjacent traces). Corresponding **(G–I)** average ECoG and **(J–L)** average speed. Each mouse is indicated by a different color; the overall average is shown in black. Gray dashed line marks the air puff onset.

**FIGURE 5:**
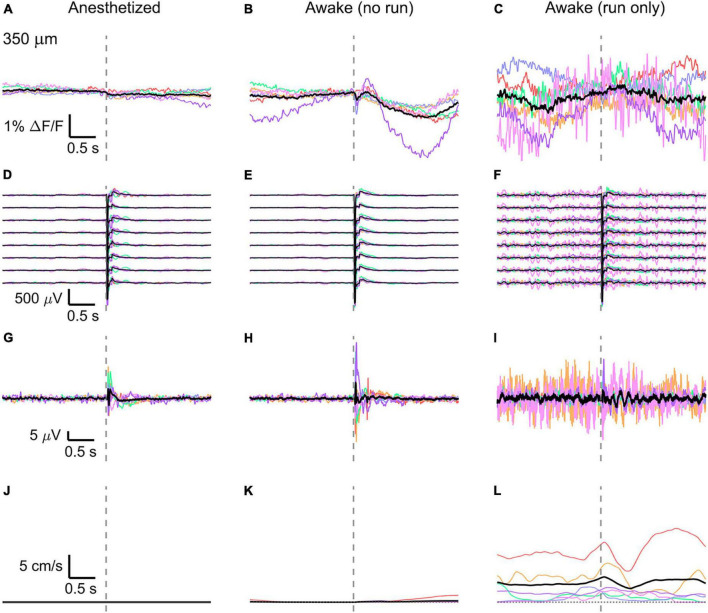
Average VSD signal 350 μm below dura and LFP, both in barrel cortex, ECoG, and running speed in response to a facial air puff (six mice). Average VSD responses evoked by an air puff to the vibrissae and cheek of the mouse in layer IV (350 μm below dura) recorded in 375 × 24 × 5 μm^3^ and 375 × 12 × 5 μm^3^ box-scans in 6 mice **(A)** during anesthesia (131, 189, 125, 138, 122, 136 trials), **(B)** in awake mice during resting (119, 174, 124, 121, 107, 112 trials), and **(C)** in awake mice during running (16, 5, 8, 12, 2, 22 trials). **(D–F)** Corresponding average LFP recorded by eight electrodes (every 4th channel of the linear probe, tip at 850 μm below dura, bottom LFP trace: 800 μm, top LFP trace: 100 μm below dura, Δdepth = 68 μm between adjacent traces). Corresponding **(G–I)** average ECoG and **(J–L)** average speed. Each mouse is indicated by a different color; the overall average is shown in black. Gray dashed line marks the air puff onset.

Then, the traces of all mice (*n* = 6) were averaged (Figures 3A–C, 4A–C, 5A–C) and compared with the average of all electrode recordings (Figures 3D–F, 4D–F, 5D–F). As expected, the shape and the amplitude of the evoked LFP response did not change when moving the objective and imaging at different cortical depths, which testify the absence of interference between the two recording modes.

With ANNINE-6plus labeling the outer leaflet of the plasma membrane, a depolarization of a cell results in a negative relative fluorescence change (Kuhn et al., 2008; Kuhn and Roome, 2019). Accordingly, the sensory stimulation causes a negative relative fluorescence change which indicates a depolarization of the average membrane potential.

In the anesthetized mouse, the optically recorded evoked voltage response was monophasic and largest in layer I and II and decreased toward deeper layers [peak amplitude (0.20 ± 0.16)%, (0.19 ± 0.16)%, (0.07 ± 0.18)% ΔF/F, Figures 3A, 4A, 5A]. The corresponding rise times (time to peak) were (265 ± 20) ms, (150 ± 9) ms, and (164 ± 14) ms, respectively.

The corresponding electrical recordings were biphasic with a sharp negative peak, lasting (33.8 ± 0.3) ms and a second, slower positive signal lasting (406 ± 13) ms (Figures 2E, 3D, 4D, 5D and Supplementary Figures 3, 4).

The amplitude of optical voltage responses in the awake resting mouse was biphasic and the first depolarization about three times higher than the ones in the anesthetized mouse [(0.65 ± 0.68)%, (0.68 ± 0.52)%, (0.33 ± 0.25)% ΔF/F, Figures 3B, 4B, 5B], lasting (197 ± 38) ms, (201 ± 21) ms, and (186 ± 15) ms respectively. Following this first, fast depolarization is a second, long lasting depolarization which is most likely due to increased neuronal or motor activity following the sensory stimulus. The mechanism at the basis of this observed trait is probably an interplay of excitatory and inhibitory circuit activity between sensory and motor areas (Manita et al., 2015). Moreover, this slower wave could be modeled by feedforward lateral inhibition, a process that is known to refine somatosensory information and helps to enhance discrimination between two different simultaneous stimulations (Cree and Weimer, 2003; Bakshi and Ghosh, 2017).

The standard deviation of the different trials before the stimulus is higher than in those recorded under anesthesia, most probably due to ongoing background activity.

The corresponding awake electrode recording shows a triphasic signal (Figures 2F, 3E, 4E, 5E and Supplementary Figures 3, 4). A fast and strong negative signal, a slower positive signal, and finally a negative signal again. Also, the electrical signal recorded in the awake state is noisier than that recorded in the anesthetized mouse.

To better evaluate the influence of running on the above results we separated the trials into run and no-run trials based on the speed of the mouse in a 500 ms window prior to the air-puff stimulation (>5 mm/s average speed for run trials, see Figures 2L,M, 3K,L, 4K,L, 5K,L). Responses acquired while the mouse was running (7–11% of trials, Figures 2D, 3C, 4C, 5C) displayed almost no peak and much higher noise. The amplitude of the first depolarization was (0.30 ± 0.74)%, (0.59 ± 0.88)%, (0.08 ± 1.39)% ΔF/F, lasting (73 ± 10) ms, (373 ± 62) ms, and (364 ± 69) ms, respectively. So, neural activity triggered in the sensory cortices by locomotion makes it harder to resolve specifically triggered sensory input, even in the stimulus onset aligned average traces.

The average running speed of the mouse often decreased following air puff stimulation (5 out of 6 mice in Figure 3L, 4 out of 6 mice in Figure 4L, 5 out 6 mice in Figure 5L). This happened most likely because the mice were not trained to the air puff stimulation, which thus represent an aversive stimulus. So, the mice tended to stop running because of this aversion to the air puff. For the sake of completeness, the speed of the mouse was computed also during anesthesia, although obviously it was always zero (Figures 2K, 3J, 4J, 5J).

The ECoG also showed an evoked response comprised in the first 500 ms after the stimulus (Figures 2H–J, 3G–I, 4G–I, 5G–I): a sharp biphasic signal (positive–negative) followed by a slower positive signal in the anesthetized mouse, which becomes an inverted biphasic signal (negative–positive) followed by a slower negative signal in the awake mouse. As seen before for the LFP recordings, the ECoG recorded during wakefulness (particularly during running) is noisier than that recorded in the anesthetized mice.

### Average Calcium and Electrical Responses

**FIGURE 6:**
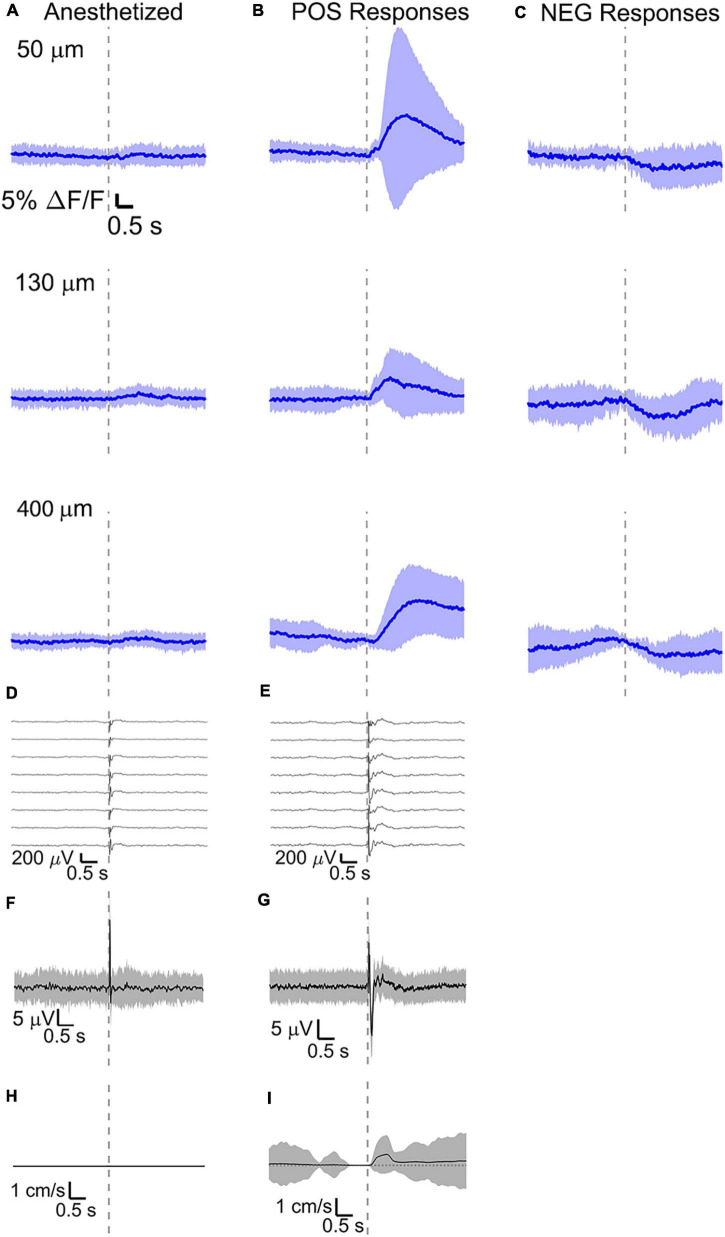
Average calcium signal at three different depth and LFP, both in barrel cortex, ECoG, and running speed in response to a facial air puff (four anesthetized and awake but resting mice). Average calcium signals recorded from three different depths in **(A)** anesthetized (4 mice, 50 μm: 236 trials, 55 ROIs, 130 μm: 221 trials, 44 ROIs, 400 μm: 228 trials, 50 ROIs) and awake but resting mice, the latter separated in **(B)** positive (4 mice, 50 μm: 213 trials, 42 ROIs; 130 μm: 227 trials, 42 ROIs, 400 μm: 215 trials, 38 ROIs) and **(C)** negative signals (4 mice, 50 μm: 213 trials, 15 ROIs; 130 μm: 227 trials, 20 ROIs, 400 μm: 215 trials, 20 ROIs). **(D,E)** Corresponding average LFP recorded by eight electrodes (every 4th channel of the linear probe, tip at 850 μm below dura, bottom LFP trace: 800 μm, top LFP trace: 100 μm below dura, Δdepth = 68 μm between adjacent traces). Corresponding **(F,G)** average ECoG and **(H,I)** average speed. All traces are plotted as mean ± standard deviation. Gray dashed line marks the air puff onset.

**FIGURE 7:**
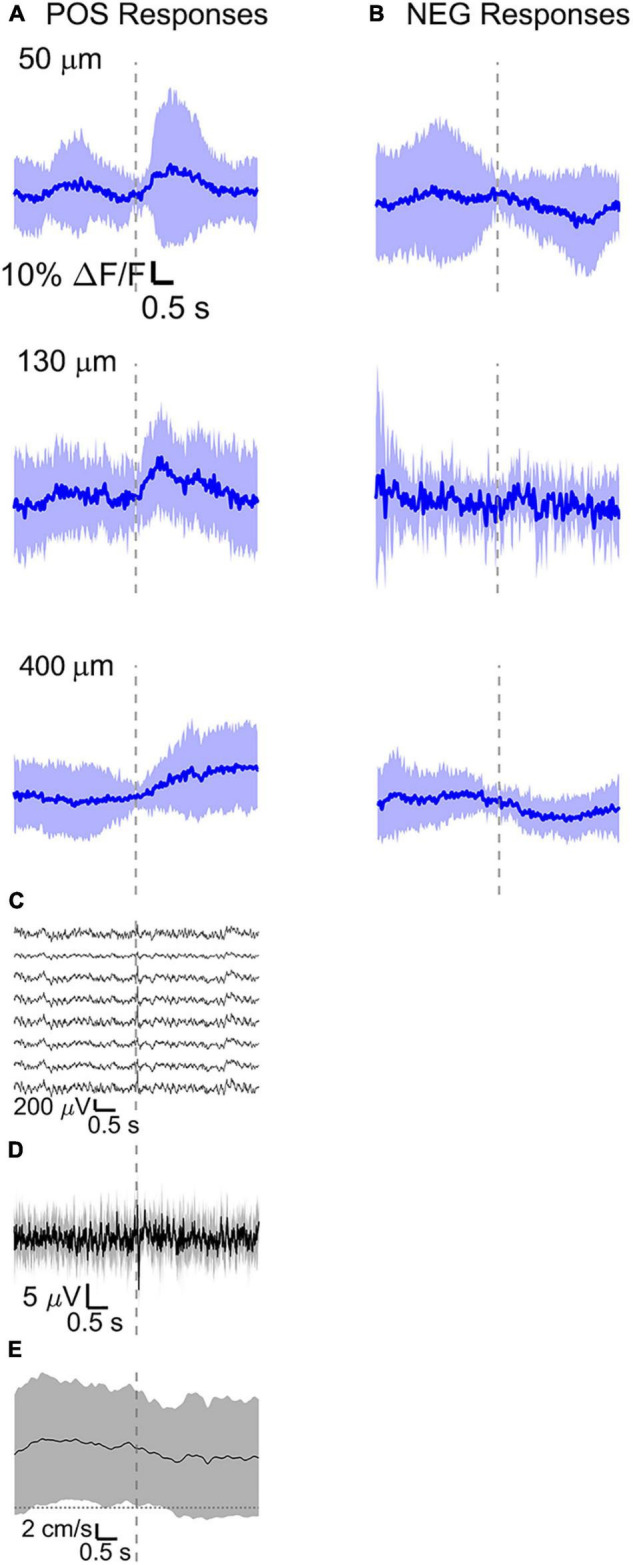
Average calcium signal at three different depth and LFP, both in barrel cortex, ECoG, and running speed in response to a facial air puff (four awake mice during running). Average calcium signals recorded from three different depths in awake mice during running, separated in **(A)** positive (4 mice, 50 μm: 24 trials, 32 ROIs; 130 μm: 9 trials, 37 ROIs; 400 μm: 25 trials, 30 ROIs) and **(B)** negative signals (4 mice, 50 μm: 24 trials, 24 ROIs, 130 μm: 9 trials, 12 ROIs, 400 μm: 25 trials, 28 ROIs). **(C)** Corresponding average LFP recorded by eight electrodes (every 4th channel of the linear probe, tip at 850 μm below dura, bottom LFP trace: 800 μm, top LFP trace: 100 μm below dura, Δdepth = 68 μm between adjacent traces. Corresponding **(D)** average ECoG and **(E)** average speed. All traces are plotted as mean ± standard deviation. Gray dashed line marks the air puff onset.

For comparison, in a separate set of experiments (*n* = 4 mice), we recorded calcium activity at the same depths with simultaneous electrical recordings. We selected somata and sorted them depending on their response to the sensory stimulus into positive and negative response types. A few best responding ROIs from a single mouse and related calcium events are shown in Supplementary Figure 5. For each mouse and each recorded depth, the average of all ROIs and all stimulus-evoked signals was computed (Supplementary Figure 3). The average across animals was then obtained and shown in Figure 6. Responses to multi-whisker stimulations recorded with GCaMP6f fluorescence were large during wakefulness, but nearly disappeared during anesthesia (Figure 6A). Evoked calcium responses acquired during wakefulness were predominantly positive (Figure 6B), but between 25 and 35% of somata showed a decrease in calcium (Figure 6C). Evoked calcium responses acquired during wakefulness were also separated in resting (Figures 6B,C,H,I) and running (Figures 7A,B,E). In contrast to the optical voltage responses discussed in “Average Optical and Electrical Voltage Responses” section, the amplitude of calcium responses during running (Figures 7A,B) was comparable to the ones during resting (Figures 6B,C).

The amplitude of average evoked LFP responses in anesthetized and awake trials is similar (Figures 6D,E) and typically of hundreds of microvolts, while the latency of the response peak is (8 ± 1) ms for the first fast peak and (72 ± 4) ms for the slower second peak. However, the second negative peak is wider in the awake mouse (lasting (140 ± 11) ms, while lasting (124 ± 8) ms in the anesthetized) and often presenting a double peak. Moreover, spontaneous activity before vibrissa stimulations seems much higher in the case of awake trials (as seen also in the previous section). LFP recordings during running do not show a clear evoked response to whisker stimulations (Figure 7C) as well as in the ECoG recording during running (Figure 7D).

## Discussion

Electrical activity is a key feature of neuronal communication and computation. Ions flow through the neuronal plasma membrane, as well as the intracellular and the extracellular space. These currents, driven by ion concentration differences, result in potential differences. Within the conducting extracellular or intracellular space, the potential differences are small because currents can easily flow and compensate the potential difference. The plasma membrane, however, is much less conducting and therefore supports a potential difference. Electrical excitation of neuronal membranes involves transient permeability increases to Na+, K+, and Ca++ ions (Hille, 1984). Here, we measure on one hand the small changes of the extracellular potential with an electrode array placed in the extracellular space. Such LFPs can be converted to current sinks and sources, indicating currents flowing into or out of cells, which can help to investigate the underlying mechanisms of neuronal activity (Mitzdorf, 1985; Buzsáki et al., 2012). On the other hand, we measure the membrane potential changes with a synthetic voltage sensitive dye molecules sitting in the plasma membrane. As the synthetic dye molecules are hydrophobic they label all membrane surfaces non-specifically–including neuron and glia cells–without penetrating the membrane. Therefore, the VSD signal reports the average plasma membrane potential changes. The LFP and the average membrane potential are tightly related to each other and still difficult to connect due to the structural complexity of the brain. Measuring them simultaneously will hopefully help to improve current biophysical models.

The here used synthetic VSD ANNINE-6plus has the advantage that the dye is purely electrochromic and responds linearly to voltage changes almost instantaneously (nanosecond time scale) (Kuhn and Roome, 2019). In this study, the unspecific labeling was desired to have a direct comparison of LFP and average membrane potential. It would be also interesting to use genetically encoded voltage indicators (Knöpfel, 2012) which allows the labeling of specific cell types and, thereby, to study their specific contributions to the LFP.

The here evoked VSD signals are in line with previous recordings made with ANNINE-6 in mice barrel cortex during anesthesia and wakefulness (Kuhn et al., 2008; Dalphin et al., 2020), showing a principal negative peak in the 500 ms following the stimulus. The response amplitudes are higher in a resting, awake mouse (Figures 3K, 4K, 5K), but decrease and almost disappear in the high neural activity triggered by running locomotion (Figures 3L, 4L, 5L).

Previously, it was shown that the majority of neurons, especially in layer 2/3, responded most strongly during the combined condition of run-with-touch (Ayaz et al., 2019). In our experiments, the main contribution to the VSD signal comes from fine dendritic processes from neurons of different layers. The signal fluctuations are much bigger than the expected sensory responses and are therefore barely visible. On the other hand, during rest, the mouse undergoes a passive stimulation. In this case whisker stimuli are expected to evoke large depolarizing sensory responses (Crochet and Petersen, 2006).

After simultaneous recording of LFP and average membrane potential, we simultaneously recorded LFP and calcium activity. Calcium ion concentration changes are often used as a proxy for electrical activity in neurons, however, calcium is a second messenger and therefore already one step down the signaling cascade. Membrane potential change result in different calcium changes in different cell types and can therefore not easily be converted. Therefore, it is important to combine the measurement of electrical and calcium activity. In the future, it would be desirable to measure LFP, average membrane potential and calcium changes at once.

Here, intracellular calcium activity showed very little response to vibrissa deflections under anesthesia (Figure 6A and Supplementary Figure 1A), while VSD imaging reported clear voltage changes in the cells of the same region. This suggests that isoflurane anesthesia may block the neuronal mechanisms in pyramidal neurons which involve major calcium influxes. This is consistent with previous findings with GCaMP6f fluorescence in anesthetized mice (Dalphin, 2019).

During wakefulness, we found that sensory stimuli can inactivate as well as enhance calcium-mediated cortical dynamics (Figures 6B,C, 7A,B and Supplementary Figures 1B,C, 2A,B), as previously observed in response to visual stimulations in layer 6 corticothalamic neurons of the mouse visual cortex (Augustinaite and Kuhn, 2020b). Negative calcium responses generated by whisker stimulations were found for a few cells at all the recorded cortical depths and in all mice, albeit with different amplitudes of the average evoked response. Moreover, negative calcium responses were found both during quiet resting and during running. Therefore, decreased evoked calcium activity seemed not connected to locomotion or a particular layer. Negative calcium responses might indicate a decrease in spiking activity and thereby a decrease in firing frequency.

Given the shallow angle of insertion of the needle probe, the electrodes locations span multiple barrels, moving along the array from layer 1 to layer 6. As the air puff activates all vibrissae similarly, we can expect that all barrels get similarly activated. Accordingly, in our experiments LFP recordings showed clear evoked responses in all electrodes.

In this methods paper, we validated the combined recording of neuronal activity *in vivo* through two-photon voltage or calcium imaging and LFP recordings in the mouse somatosensory cortex, confirming the possibility of using the two techniques simultaneously with highly reduced optical noise and no photo-induced interferences in the electrical recordings.

We envision that this platform could be used and expanded in many awake and behavioral experiments targeting different brain regions, possibly with chronically implanted neuro probes and flexible connectors. Flexible layouts can serve to mechanically decouple the needle probe from the connectors, thus limiting the problems related to the steric hindrances of the setups and making it easier to perform repeated experiments on the same animal.

Most importantly, we hope our dataset will help theorists to explain how current sinks and sources connect measured LFP signals to average membrane potential changes measured with the pure electrochromic VSD ANNINE-6plus, and how these voltage and current changes trigger calcium changes.

## Data Availability Statement

The datasets presented in this study can be found in online repositories. The names of the repository/repositories and accession number(s) can be found below: doi: 10.5061/dryad.dbrv15f23.

## Ethics Statement

The animal study was reviewed and approved by OIST Institutional Animal Care and Use Committee (IACUC).

## Author Contributions

CC and BK designed the experiments. CC performed the experiments and analyzed the data. All authors wrote the manuscript.

## Conflict of Interest

The authors declare that the research was conducted in the absence of any commercial or financial relationships that could be construed as a potential conflict of interest.

## Publisher’s Note

All claims expressed in this article are solely those of the authors and do not necessarily represent those of their affiliated organizations, or those of the publisher, the editors and the reviewers. Any product that may be evaluated in this article, or claim that may be made by its manufacturer, is not guaranteed or endorsed by the publisher.

## Footnotes

1
http://atlasneuro.com

2
http://www.sensitivefarbstoffe.de

## Supplementary Material

Supplementary Figure 1 Average calcium responses in barrel cortex in response to air puff stimulation in anesthetized **(A)** and awake but resting mice at three different cortical depths, separated in **(B)** positive and **(C)** negative signals. Responses are plotted as averages over: for anesthetized, 50 μm: 70, 57, 54, 55 trials; 130 μm: 58, 58, 52, 53 trials; 400 μm: 62, 59, 50, 57 trials; for awake resting, 50 μm: 59, 51, 50, 53 trials; 130 μm: 64, 60, 45, 58 trials, 400 μm: 62, 56, 48, 49 trials. Every color represents one mouse. The gray dashed line indicates the air puff onset.

Supplementary Figure 2 Average calcium responses in barrel cortex in response to air puff stimulation in awake mice during running at three different cortical depths, separated in **(A)** positive and **(B)** negative signals. Responses are plotted as averages over: 50 μm: 5, 6, 9, 4 trials; 130 μm: 0 (no running traces), 2, 6, 1 trials, 400 μm: 3, 3, 13, 6 trials. Every color represents one mouse. The gray dashed line indicates the air puff onset.

Supplementary Figure 3 Zoom in on average LFP responses to vibrissa stimulation of Figure 2.

Supplementary Figure 4 Zoom in on average LFP responses to whisker stimulation of Figures 3–5.

Supplementary Figure 5 Representative 375 × 375 μm^2^ field of view for calcium imaging and calcium transients of few selected most responsive cells during the experimental conditions in response to air puff stimulation. **(A)** 50 μm (layer I), **(B)** 130 μm (layer II), **(C)** 400 μm (layer IV).
