# Supplementary figures and images for: Simultaneous Two-Photon Voltage or Calcium Imaging and Multi-Channel Local Field Potential Recordings in Barrel Cortex of Awake and Anesthetized Mice
Source: Front Neurosci. 2021 Nov 11;15:741279. doi: 10.3389/fnins.2021.741279 (PMC8632658; doi:10.3389/fnins.2021.741279)

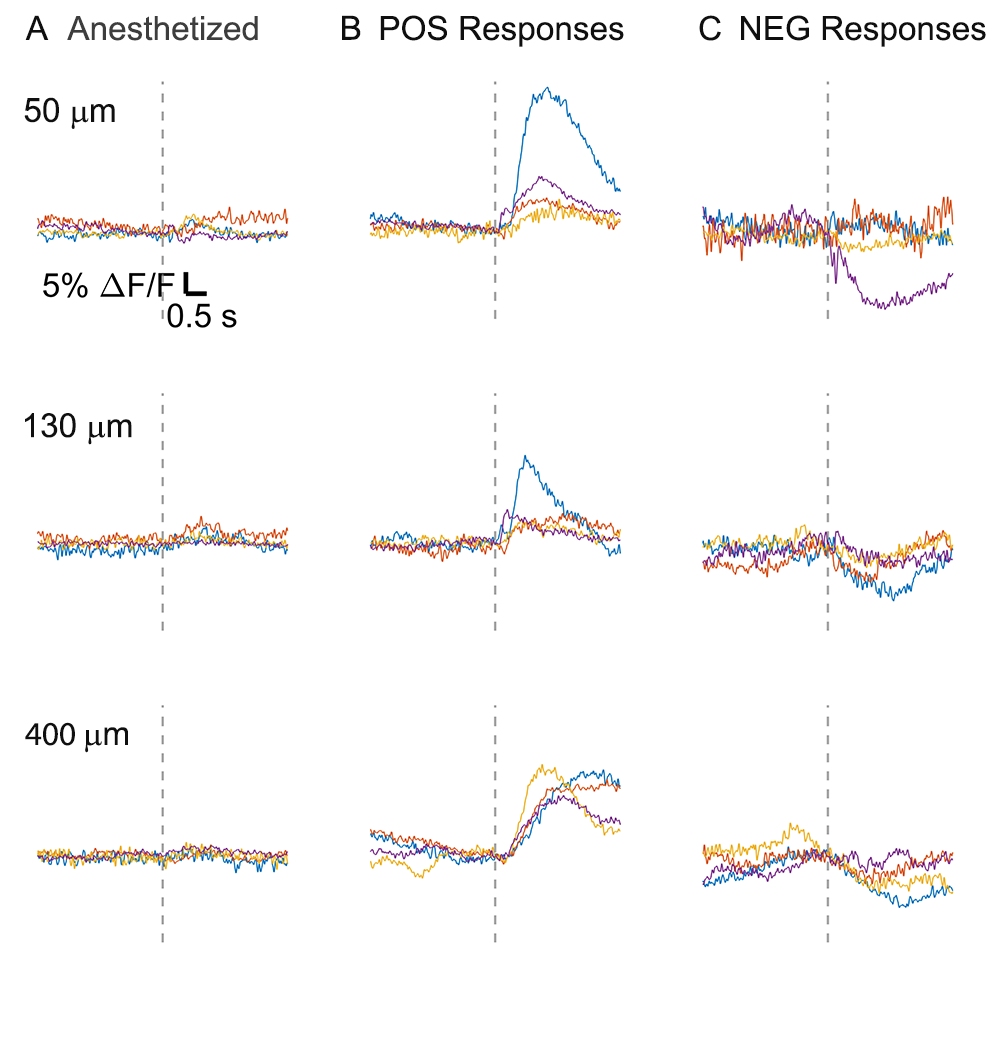

Supplement: Supplementary Figure 1 — Average calcium responses in barrel cortex in response to air puff stimulation in anesthetized (A) and awake but resting mice at three different cortical depths, separated in (B) positive and (C) negative signals. Responses are plotted as averages over: for anesthetized, 50 μm: 70, 57, 54, 55 trials; 130 μm: 58, 58, 52, 53 trials; 400 μm: 62, 59, 50, 57 trials; for awake resting, 50 μm: 59, 51, 50, 53 trials; 130 μm: 64, 60, 45, 58 trials, 400 μm: 62, 56, 48, 49 trials. Every color represents one mouse. The gray dashed line indicates the air puff onset. [file Image_1.TIF]

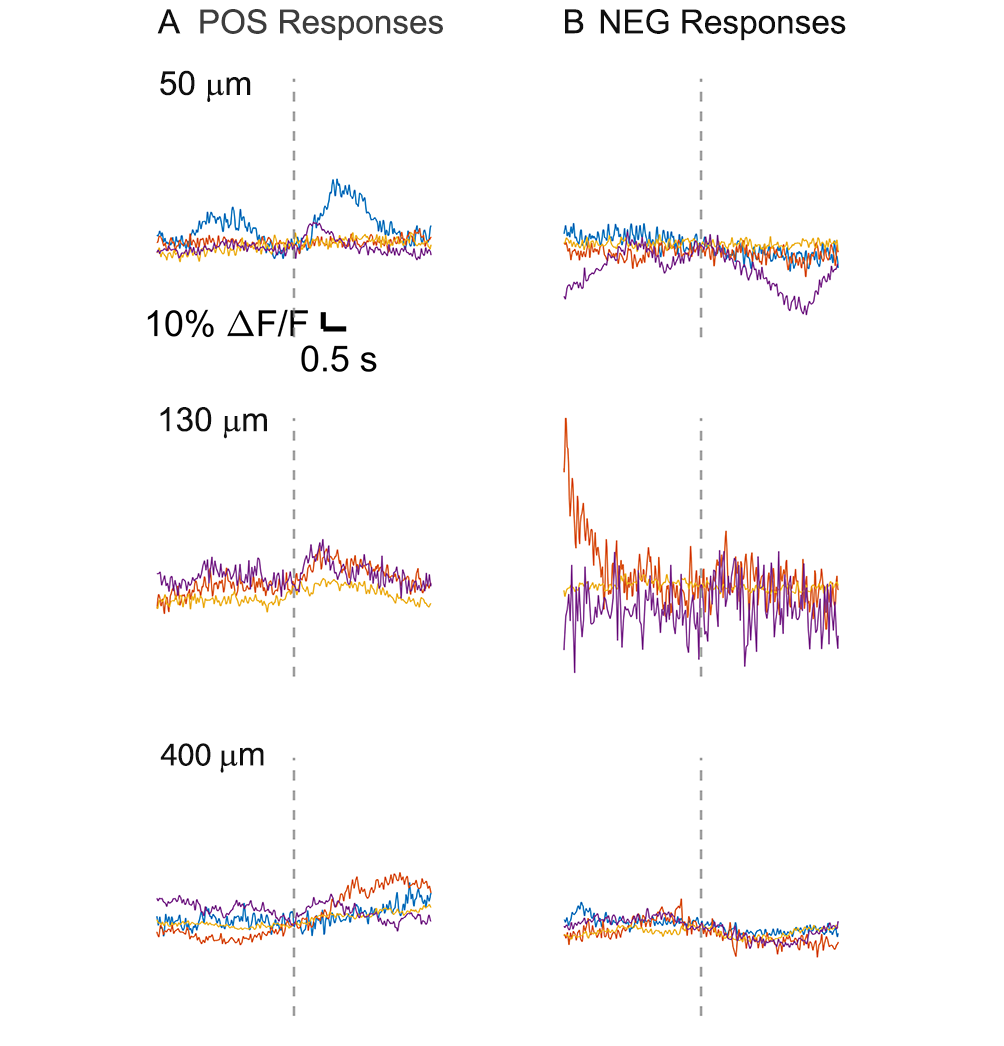

Supplement: Supplementary Figure 2 — Average calcium responses in barrel cortex in response to air puff stimulation in awake mice during running at three different cortical depths, separated in (A) positive and (B) negative signals. Responses are plotted as averages over: 50 μm: 5, 6, 9, 4 trials; 130 μm: 0 (no running traces), 2, 6, 1 trials, 400 μm: 3, 3, 13, 6 trials. Every color represents one mouse. The gray dashed line indicates the air puff onset. [file Image_2.TIF]

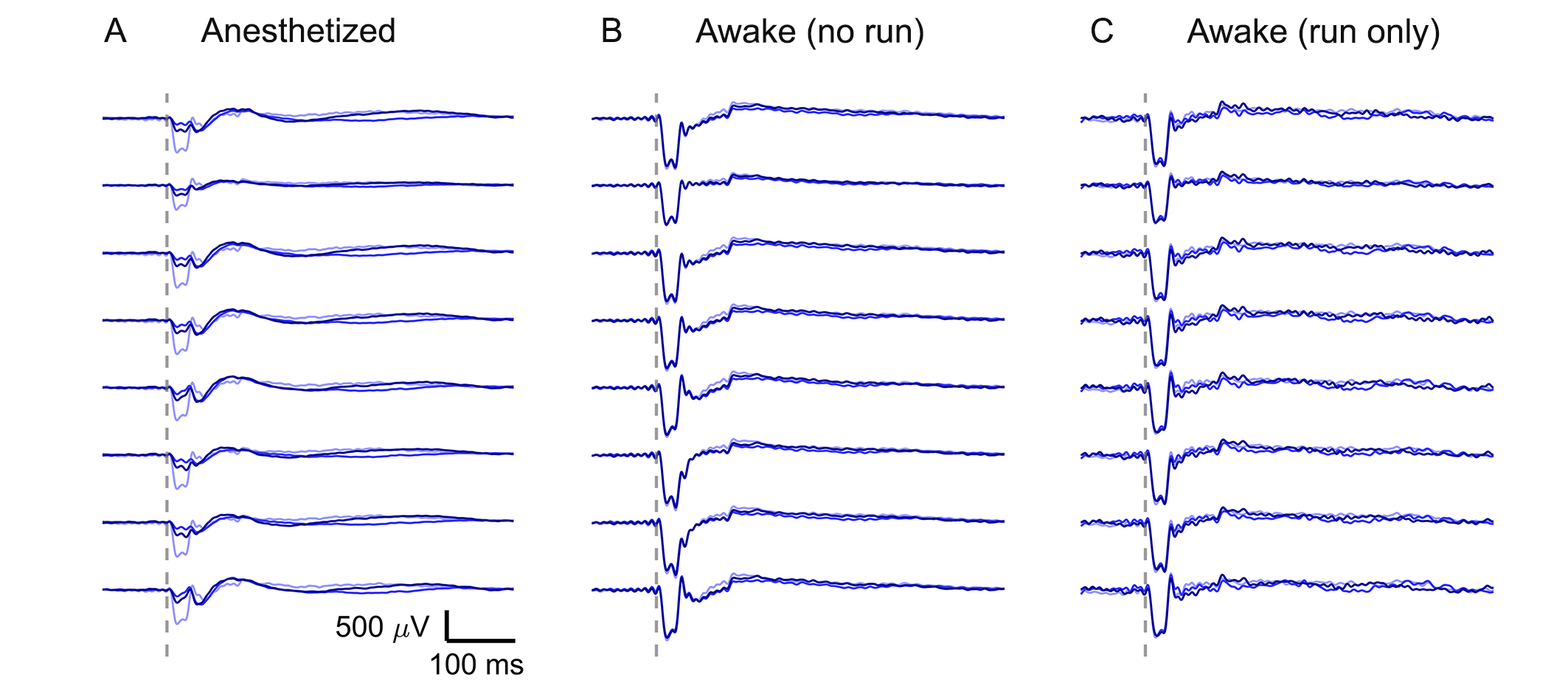

Supplement: Supplementary Figure 3 — Zoom in on average LFP responses to vibrissa stimulation of Figure 2. [file Image_3.TIF]

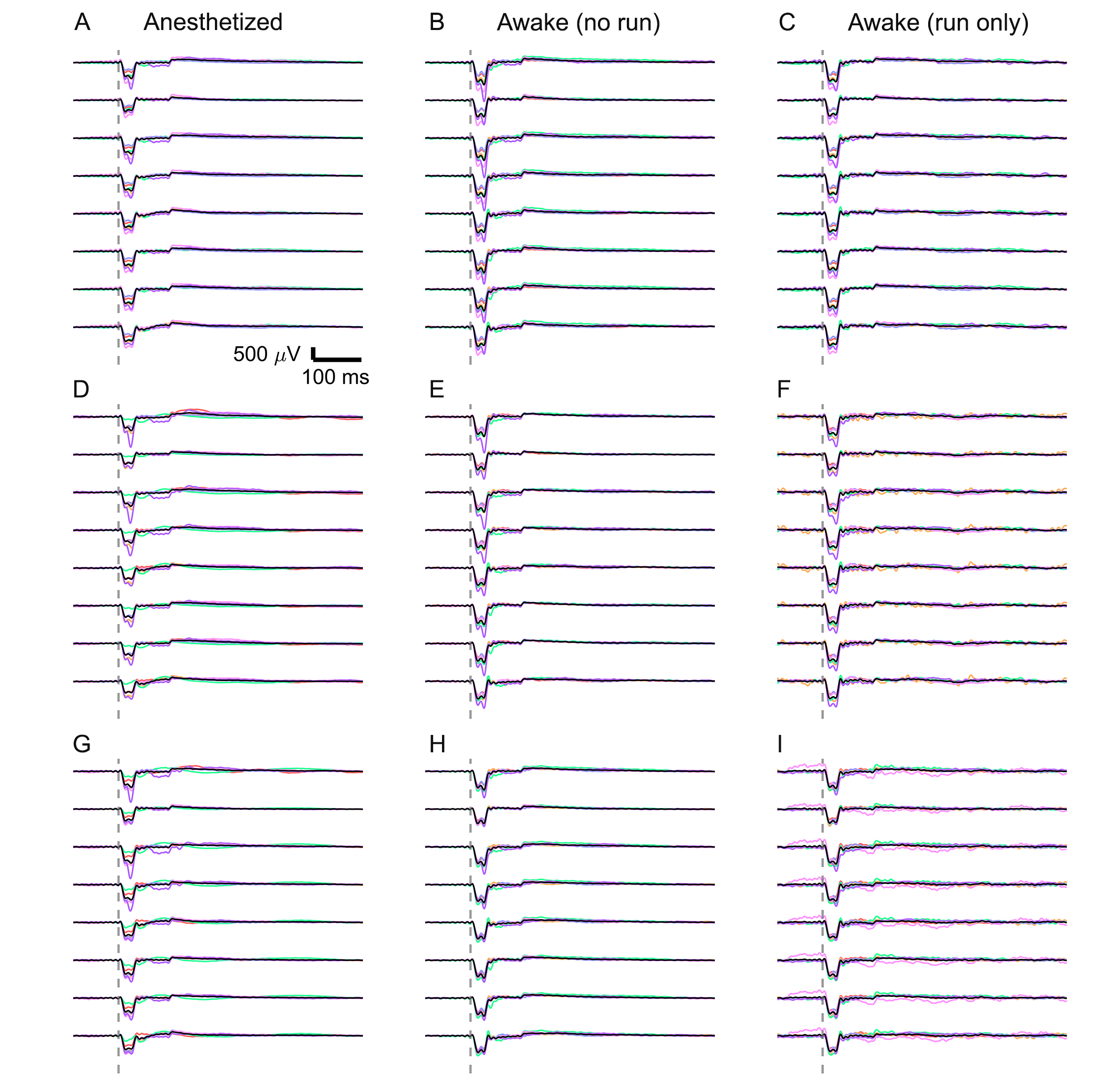

Supplement: Supplementary Figure 4 — Zoom in on average LFP responses to whisker stimulation of Figures 3–5. [file Image_4.TIF]

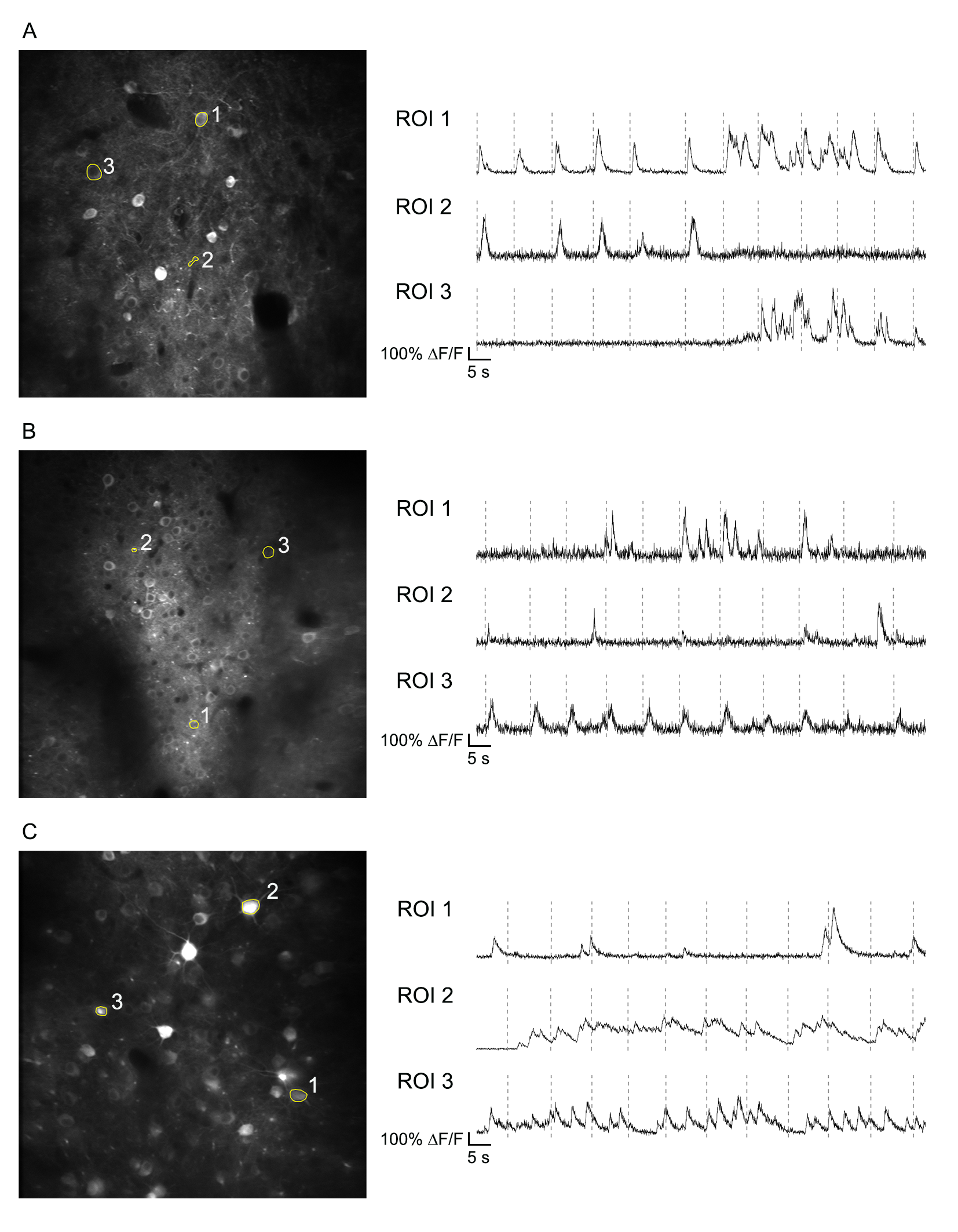

Supplement: Supplementary Figure 5 — Representative 375 × 375 μm2 field of view for calcium imaging and calcium transients of few selected most responsive cells during the experimental conditions in response to air puff stimulation. (A) 50 μm (layer I), (B) 130 μm (layer II), (C) 400 μm (layer IV). [file Image_5.TIF]
